# The autologous chondral platelet-rich plasma matrix implantation. A new therapy in cartilage repair and regeneration: macroscopic and biomechanical study in an experimental sheep model

**DOI:** 10.3389/fvets.2023.1223825

**Published:** 2023-12-11

**Authors:** Pau Peláez-Gorrea, Elena Damiá-Giménez, Mónica Rubio-Zaragoza, Belén Cuervo-Serrato, Ángel María Hernández-Guerra, Laura Miguel-Pastor, Ayla Del Romero-Martínez, Joaquín Sopena-Juncosa, Marta Torres-Torrillas, Angelo Santana, Ramón Cugat-Bertomeu, José Manuel Vilar-Guereño, Jose Maria Carrillo-Poveda

**Affiliations:** ^1^Bioregenerative Medicine and Applied Surgery Research Group, Department of Animal Medicine and Surgery, CEU-Cardenal Herrera University, CEU Universities, Valencia, Spain; ^2^García Cugat Foundation CEU-UCH Chair of Medicine and Regenerative Surgery, CEU-Cardenal Herrera University, CEU Universities, Valencia, Spain; ^3^Departament of Mathematics, Universidad de Las Palmas de Gran Canaria, Las Palmas, Spain; ^4^Department of Animal Pathology, Instituto Universitario de Investigaciones Biomédicas y Sanitarias, University of Las Palmas de Gran Canaria, Las Palmas de Gran Canaria, Spain

**Keywords:** cartilage regeneration, regenerative medicine, plasma rich in growth factors, autologous platelet rich plasma matrix implantation, force platform, symmetry index

## Abstract

**Introduction:**

Articular cartilage injuries are a severe problem, and the treatments for these injuries are complex. The present study investigates a treatment for full-thickness cartilage defects called Autologous Chondral Platelet Rich Plasma Matrix Implantation (PACI) in a sheep model.

**Methods:**

Chondral defects 8 mm in diameter were surgically induced in the medial femoral condyles of both stifles in eight healthy sheep. Right stifles were treated with PACI and an intraarticular injection with a plasma rich in growth factors (PRGF) solution [treatment group (TRT)], while an intraarticular injection of Ringer’s lactate solution was administered in left stifles [Control group (CT)]. The limbs’ function was objectively assessed with a force platform to obtain the symmetry index, comparing both groups. After 9 and 18 months, the lesions were macroscopically evaluated using the International Cartilage Repair Society and Goebel scales.

**Results:**

Regarding the symmetry index, the TRT group obtained results similar to those of healthy limbs at 9 and 18 months after treatment. Regarding the macroscopic assessment, the values obtained by the TRT group were very close to those of normal cartilage and superior to those obtained by the CT group at 9 months.

**Conclusion:**

This new bioregenerative treatment modality can regenerate hyaline articular cartilage. High functional outcomes have been reported, together with a good quality repair tissue in sheep. Therefore, PACI treatment might be a good therapeutic option for full-thickness chondral lesions.

## Introduction

Chondral lesions are a frequent source of knee discomfort and dysfunction. Moreover, articular cartilage lesions can lead to a premature onset of Osteoarthrosis (OA), having a vastly negative impact on patient’s functionality and quality of life (1). The reparative and regenerative capacity of the cartilage is limited because of the absence of blood vessels in the tissue and the inability of chondrocytes to react to tissue damage (2). During the articular cartilage repair process, the newly formed tissue has a fibrous nature; thus, the biomechanical properties of native hyaline cartilage are lost, leading to articular degeneration (3). Several strategies to treat articular cartilage injuries have been proposed, including some palliative techniques like chondroplasty and debridement, reparative strategies such as microfractures, or restorative treatments such as mosaicplasty (osteochondral autograft transfer), osteochondral allograft transplantation, scaffold-based repair (in combination or not with cell therapy), autologous chondrocyte implantation (ACI), or matrix-induced ACI (4, 5). The main limitations of these techniques include fibrocartilage formation without long-lasting improvement, the high cost of the treatments, the need for two surgical procedures, longer recovery times, or variable results in active patients (2, 5). For these reasons, further research is needed to find therapies that improve the actual treatments of cartilage injuries (1).

The use of platelet-rich plasma (PRP) and its derivatives have been shown to enhance regeneration in multiple tissues. Many studies show that growth factors (GFs) within the PRP can lead to an increase in the biosynthesis of the cartilage matrix proteins, stimulate chondrogenic regeneration, and improve chondrocyte proliferation and metabolism (2). Moreover, PRP also contains several proteins (fibrinogen, fibronectin, and vitronectin) that play a key role in tissue repair and regeneration. They enable cell and other molecules adhesion, allow cell conduction, and behave as a support “matrix” for tissue repair (6). It has been demonstrated that when an intraarticular (IA) injection of PRP is inserted into the joint, a three-dimensional network of fibrin containing binding sites is created to enhance cellular adhesion, creating a microenvironment that stimulates the cartilage repair process (7). Another bioproduct is Platelet-Poor Plasma (PPP), which has a lower concentration of platelets than PRP. However, it was proven that PPP accelerates cell migration and facilitates healing-associated cell functions (8). There are different types of PRP depending on the preparation protocol and its content. Plasma Rich in Growth Factors (PRGF) is a type of PRP elaborated by a single centrifugation process that is characterized for being 100% autologous and biocompatible. Additionally, it has a moderate platelet concentration, and it is free of leukocytes and erythrocytes (9, 10). It has an autologous origin; thus, it is considered a safe product with a low incidence of reported adverse effects, which makes it a suitable treatment for patients with a chondral lesion, especially when other therapeutic options are not recommended (11, 12).

Recently, a novel autologous-made matrix called Autologous Chondral PRP Matrix Implantation (PACI) has been used for the treatment of chondral lesion instead of other techniques (4, 5, 13). PACI consists of hyaline cartilage chips associated with a clot of PRP and PPP in combination with IA PRGF in liquid stage. This has already been assessed for the treatment of full-thickness cartilage and osteochondral knee defects in humans (14, 15) and sheep (2, 16, 17). The novel technique harvests cartilage tissue from the chondral defect and combines it with PRP scaffolds. PRP scaffold works as a bioactive component that interacts with embedded cartilage fragments, inducing chondrocytes migration, proliferation, and differentiation to ease cartilage repair. This therapy provided satisfactory functional results and pain relief, with excellent quantity and quality of the repaired tissue (14, 15). Next, the PRP derivative is used as an adjuvant, instead of a main treatment, in these cases. Along the same line, Dominguez et al. performed a preliminary study in sheep to evaluate the efficacy of PACI in stifle chondral lesions, evaluating the histology and the immunohistochemistry of the repaired tissue to discern the type of collagen (type I or II) and the proportional amount that can be found within the implant (2). This aspect is crucial because type II collagen is the main component of healthy cartilage (90–95%) (18), while the presence of type I collagen in articular cartilage indicates the existence of fibrotic connective tissue (19). Similarly, Alcaide-Ruggiero et al. evaluated the macroscopic appearance, histological structure, and chondrocyte repair in cartilage defects in sheep, and the PACI+PRP groups showed significantly better results in the percentage of defect repair and chondrocytes in the newly formed cartilage tissue at 18 months compared to 9 months (17). Recently, it has been published that the PACI+PRP treatment improves the repair cartilage process in chondral defects with mature hyaline cartilage. It also enhances the structural and mechanical qualities of the repair tissue, achieving a more consistent cartilage that is less susceptible to degradation and to producing hypertrophic formations over time (16). The results of the studies showed that PACI is an optimal technique to repair hyaline cartilage, with an adequate presence of type II collagen and minimal presence of type I collagen (2). Moreover, studies in humans have demonstrated that PACI is a reliable and competent surgical procedure with promising results (15).

Lameness is an obvious manifestation of musculoskeletal disorders (20). Force platform gait analysis is considered the “gold standard” for the objective evaluation of limb function or to evaluate changes related to musculoskeletal limb diseases (21). Gait and movement asymmetries are significant variables for measuring locomotor biomechanics in patients; furthermore, these variables play a key role in the prediction of future injuries or in assessing the success of clinical interventions. The Symmetry Index (SI) is the measure most often used, where the denominator is taken to be the average of the absolute values for both limbs. SI is the ratio of the gait cycle between the limbs to compare the dominance pattern between study groups (22). To calculate the SI, it is necessary to obtain the ground reaction force data, including the peak vertical force (PVF), and the vertical impulse (VI) (23). Furthermore, the SI measures the degree of symmetry between different limbs and can be used to determine if a patient is clinically sound or lame (24). If the SI is greater than 3%, then it means the animal is lame, while a SI lower than 3% indicates the animal supports the same force on both limbs and is sound (25).

To macroscopically assess the repair of the chondral lesion, the ICRS and Goebel scales are used. The ICRS scale assesses the degree of defect repair, the integration of the border zone, and the general macroscopic appearance of the defect. The ICRS scale defect is scored between 0 (the worst possible result) to 12 (the best possible result) (26). On the other hand, the Goebel scale score uses five major parameters: “the color of the repair tissue,” “the presence of blood vessels in the repair tissue,” “the surface of the repair tissue,” “the filling of the defect,” and “the degeneration of adjacent articular cartilage.” For the Goebel scale, the defect is scored between 0 (excellent repaired tissue) to 20 (no repaired tissue and damaged adjacent area).

The purpose of this study is to evaluate the functional therapeutic potential of PACI for the treatment of stifle full-thickness cartilage defects using a force platform and a macroscopical evaluation in a sheep model. It was hypothesized that this technique would repair articular cartilage, and clinical, biomechanical, and macroscopical improvements would be obtained.

## Materials and methods

### Animals

The present study was approved by the Bioethical Committee for Animal Experimentation of the CEU-Cardenal Herrera University (2019/VSC/PEA/0162 type 2), and it has strictly complied with the experimental animal legislation adhered to by the Ministry of Agriculture, Fisheries and Food. In the study group, eight skeletally mature Merino sheep with a mean weight of 52 kg were included. An additional four sheep were used as a negative control group. The animals were exhaustively evaluated by a veterinarian to ensure an optimal healthy status and to rule out any orthopedic problems.

### Study groups

A full-thickness chondral defect 8 mm in diameter was created in the medial femoral condyles of both stifles in all animals. The right stifles were treated with PACI combined with an IA injection of PRGF (TRT stifles), and the left stifles were treated with a single IA injection of Ringer Lactate solution (RLS; CT stifles).

### Presurgical procedure

Before surgery, the animals fasted for 24 h to prevent possible complications caused by regurgitation and tympany during the procedure and recovery. All sheep were premedicated with 4 mcg/kg IV Dexmedetomidine (Dexdomitor; Orion Pharma, Spain) and 0.2 mg/kg IV Morphine (Morphine 2%; B. Braun). Subsequently, animals were preoxygenated by mask at 5 L/min for a period of 5 min, followed by induction with 1 mg/kg IV Propofol (Propofol Lipide 1%; B. Braun) per minute until endotracheal intubation was performed. Moreover, esophageal catheterization was necessary to avoid ruminal tympany during anesthesia. Anesthetic maintenance was mask-induced and Sevoflurane (Sevoflo; Zoetis Belgium SA) was vaporized in 100% O_2_ (IsoTec 5; Datex-Ohmeda, United Kingdom) through a circular air system (S/5 Avance; Datex-Ohmeda anesthesia equipment, Finland).

Patient monitoring was achieved using a multiparametric vital signs monitor (S/5 Datex-Ohmeda) that consisted of an electrocardiogram, a pulsometer, a capnography, body temperature, and invasive blood pressure.

## After aseptic surgical field preparation, surgery was performed in both stifles

### Surgical procedure

**Figure 1:**
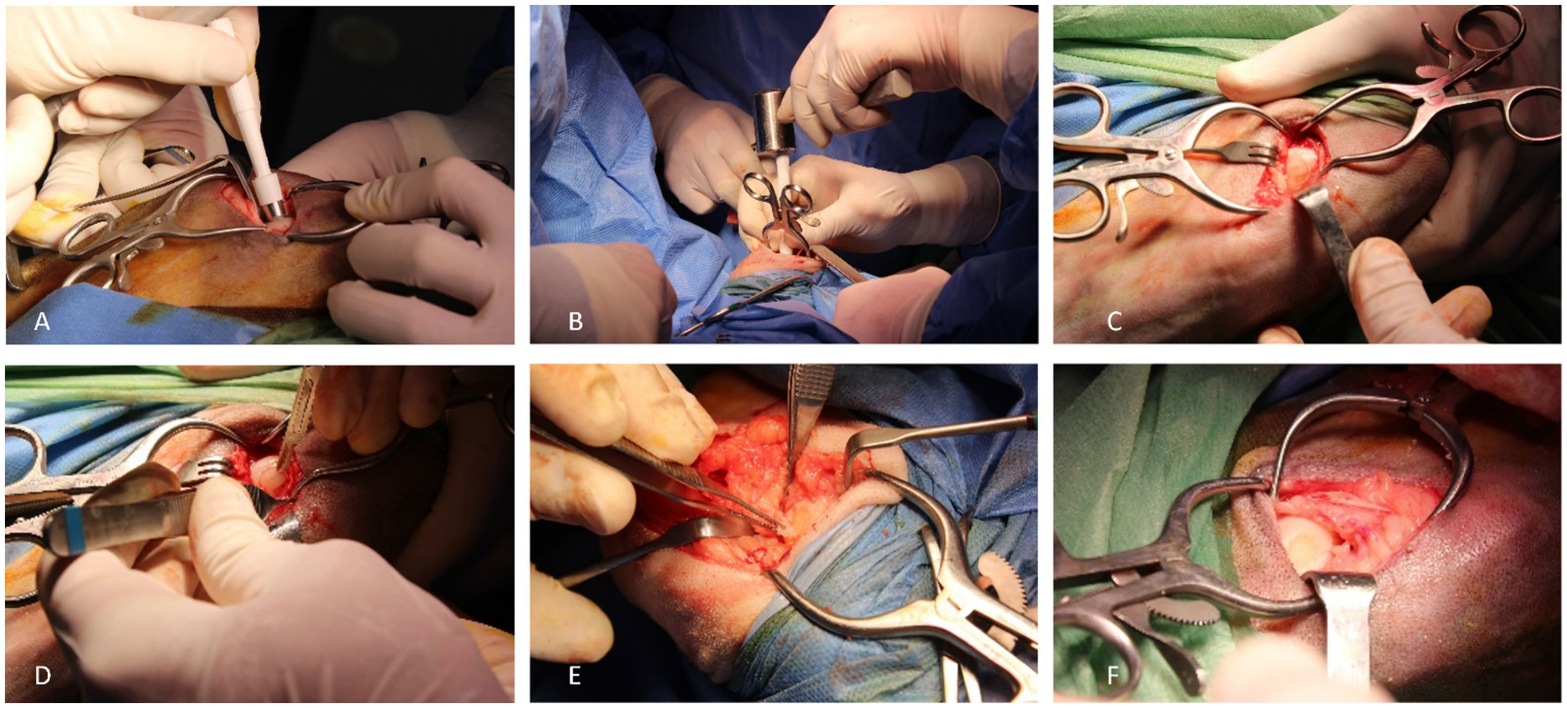
Surgical procedure. 8 mm punch placement in the load area in medial femoral condyle **(A)**; delimitation of the defect area by using a punch and a surgical hammer **(B)**; delimitated cartilage area for posterior removal **(C)**; cartilage removal by using a number 15 blade scalpel **(D)**; articular cartilage removal **(E)**; created defect **(F)**.

**Figure 2:**
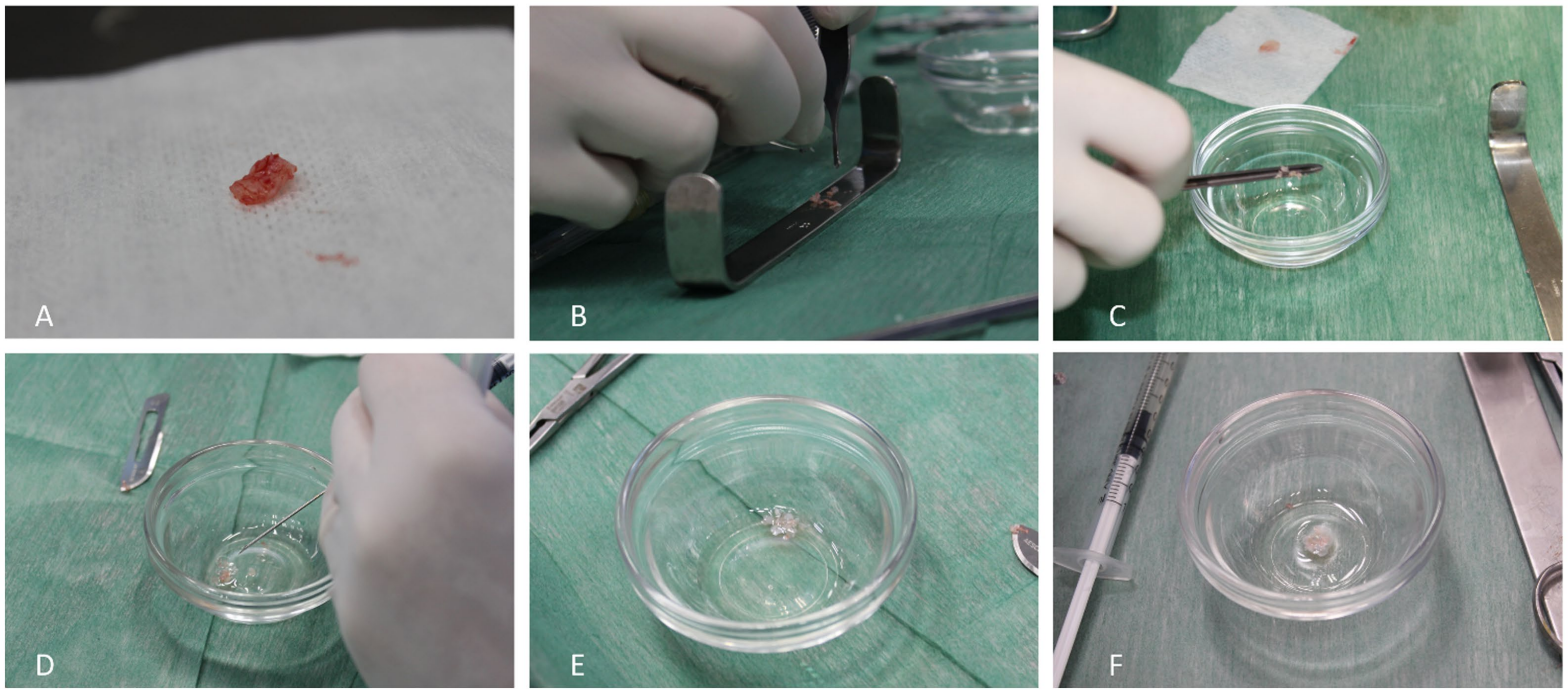
PACI creation. Cartilage fragment from the loading area of the medial femoral condyle **(A)**; fragmentation of the articular cartilage into small pieces (1-2 mm^3^) **(B)**; PACI formation into a punt **(C)**; minced cartilage together with the activated PRP **(D)**; PRP gelification **(E)**; obtained PACI **(F)**.

**Figure 3:**
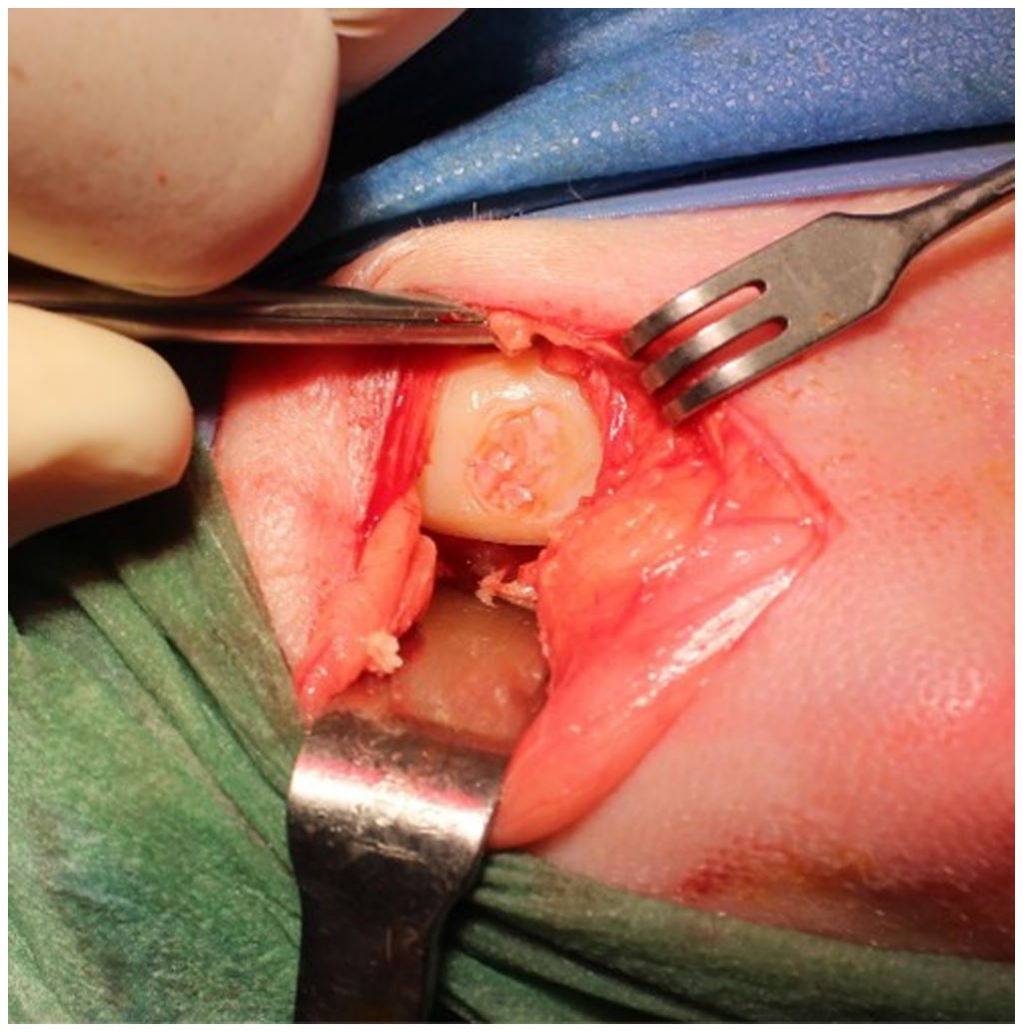
Placement of PACI in the chondral lesion of the medial condyle of the right stifle.

A 3–4 cm mini-arthrotomy was performed after medial parapatellar approach. Thereafter, the weight bearing area of the medial femoral condyle was identified and the defect was created using an 8-mm diameter punch. Subsequently, this cartilage area was removed using a number 15 scalpel blade (Figure 1). The healthy hyaline cartilage samples were then cut into small fragments (1–2 mm^3^) and mixed with the activated PRP and PPP to obtain a clot. This clot worked as a scaffold for the cartilage chips (Figure 2). Once the PACI+PRP matrix was solidified, it was placed, filling the cartilage defect (Figure 3).

The right knees of all individuals were treated with PACI. Just 5 to 10 min after PACI application on the defect, the joint was subjected to passive flexion and extension movements to ensure biological adhesion of the therapy. Subsequently, conventional closure was performed with a simple continuous suture pattern of the joint capsule with 0 polygluconate (Monosyn^®^, B Braun).

In the left knee, the lesion was created following the same procedure, but no treatment was applied.

Then, all animals received a single 2 mL IA injection of PRGF on the right joints, while the left joints were IA injected with 2 mL of RLS. Antibiotherapy (Amoxicillin 15 mg/kg IM, Amoxicilina 15%, Virbac, Spain) and analgesia (Buprenorphine 0.02 mg/kg IM, Buprecare 0.3 mg/mL, Ecuphar, Spain) were given during a 5-day period.

After anesthesia recovery, animals were housed in species-appropriate facilities without movement restriction. No casts, bandages, or splints were used.

### Autologous chondral platelet-rich plasma matrix implantation and plasma rich in growth factors: preparation and use

Before the surgical procedure, a total of 36 mL of blood was collected from the right jugular vein of each animal with a 16 g sterile needle for PRGF preparation and for blood testing to ensure the animal’s healthy status.

PRGF was prepared following PRGF Endoret^®^ methodology (Biotechnology Institute, Vitoria, Spain) and considering the data of the preliminary study (2). 25 mL of blood was collected from the jugular vein of each animal just prior surgery into 5 mL sodium citrate solution (3.8%) extraction tubes. The different blood phases were separated by centrifuging the tubes for 8 min at 630 g. The fraction located just above the buffy coat (white fraction) corresponded to PRGF; moreover, another fraction located just below the PRGF corresponded to PPP, which has demonstrated clinical efficacy (1, 2). Prior to infiltration and clot preparation, the platelets were activated using calcium chloride 10% (CaCl_2_ 10%) at a rate of 50 μL per milliliter of plasma, and the tubes were kept at room temperature (27).

The activated PPP and PRP fractions (50/50 ratio) were collected and used to prepare the clot together with the cartilage chips obtained during the surgical procedure with the 8 mm punch, creating the PACI regenerative therapy. A mean time of less than 1 h elapsed between blood collection to PRP and PPP application. A single treatment with growth factors was intraoperatively applied (2).

### Force platform

Animals were evaluated 1, 3, 9, and 18 months after surgery using a force platform. Once the PVF in the TRT and CT stifles was obtained, reference values were also obtained in healthy stifles (HS). Then, the SI for each animal was obtained. For data recording, a force platform was placed at the center of a 6 m runway covered by a rubber mat, and the sheep were forced to walk over it. Walking speed was measured by a motion sensor (PS-2103a, Pasco, CA, United States). To ensure the homogeneity of the samples, only the samples recorded within a narrow variation of velocity (1.6 ± 0.5 m/s) and acceleration (≤0.5 m/s2) were considered valid. In addition, the limbs must have contacted the center of the force platform during recording to be considered valid. Three valid trials per sheep were obtained. The sampling frequency was set to 250 Hz. The software DataStudio^®^ (Pasco, CA, United States) was used for the acquisition, numerical conversion, and storage of data.

The TRT and CT stifles were compared by means of the use of the SI. The following formula to obtain the SI was used:


$$SI=200\;\left(\mathrm{VTRT}-VCT\right)/\left(\mathrm{VTRT}+VCT\right)$$

where V_TRT_ is the PVF value in the limb PACI and V_CT_ is the PVF value of the variable measured in the limb RLS.

### Macroscopic evaluation

Animals were euthanized at 9 and 18 months (four animals at each study time) post-surgery to evaluate the repaired tissue. Digital high-resolution photographs of the articular surface of the medial femoral condyle were taken. The macroscopic repair process was assessed by four blinded observers. Two different scales were used: the validated ICRS (26) scale and a scoring system developed and validated in a sheep model in 2012 by Goebel et al. (28). The ICRS and Goebel scales were assessed in four sheep at month 9 and the remaining four at month 18.

### Statistical study

The sample size was calculated by a power analysis consistent with results published in prior research conducted by Milano et al. (29). An alpha level of 0.05 and a power of 80% were established. The following equation was used:


$$n=\frac{2\left(Z_{\alpha }+Z_{\beta }\right)2*S2}{d2}$$

For the statistical analysis of data, a linear mixed effects model has been used (30, 31). For each response variable (TRT, CT, and SI), the month has been considered as the fixed effects factor, while the sheep is a random effects factor. The model is as follows:


$$y_{ij}=\mu_{i}+b_{j}+\varepsilon_{ij},\;i=0,1,3,9,18,\;j=1,\ldots ,8$$

$$b_{i}\approx N\left(0,\sigma_{b}\right)\;\varepsilon_{ij}\approx N\left(0,\sigma \right)$$

where:

$y_{ij}$ : is the measure in the *i-th* month ( i=0,1,3,9,18 ) on the sheep j ( j=1…8 ).

$\mu_{i}$ is the (fixed) effect of the month i . This parameter represents the mean value of the variable that month.

$b_{j}$ is the (random) effect of sheep j. Values of $b_{j}$ are supposed to be normally distributed with a mean of 0 and a standard deviation of $\sigma_{b}$ . So, $\sigma_{b}$ is the variability in the response due to the sheep.

$\varepsilon_{ij}$ is the residual in the measure ij . This variable is assumed to be also normally distributed with the mean 0 and the standard deviation σ .

Parameters in this model are estimated by using the package nlme in the **R** statistical software (The R Project for Statistical Computing). The Tukey type post-hoc test was applied for locating significant differences when the overall test is significant. The Shapiro–Wilk test was used to assess the normality of the residuals, and the Levene test was applied to evaluate homoscedasticity.

Regarding the macroscopical assessment, the data were analyzed using linear mixed effects models. The “limb” and “month” were considered fixed effects, and the “sheep” and “observer” were considered random effects. The response variable was transformed using a Box-Cox transformation when necessary to achieve the normality of the residuals. The normality of the residuals was tested by the Shapiro–Wilk test. Results are shown as adjusted means (±SD) for each month, stifle, and scale, and as differences between stifles or between months, with corresponding 95% confidence intervals (CI). The significance of the differences has been assessed by type III analysis of variance, using the Satterthwaite method to adjust the degrees of freedom. Differences with *p*-values of less than 0.05 were considered significant.

To evaluate these results, we have taken adjusted mean values per limb, and they are shown for an “average” observer and an “average” sheep. The concept of adjusted mean has to do with the way the model has been estimated to analyze the data. The limb has been considered a fixed effect, one limb has treatment and the other does not, and the objective of the study is to compare the PACI treatment with the RLS control. On the other hand, the observer and the sheep are considered random effects. The model estimates the variability between scores due to having different observers and different sheep, and it then estimates the score that would have been given by an “average observer” to an “average sheep.”

## Results

### Force platform

Tables 1, 2 show the differences between the mean value of TRT limbs and CT limbs at each study time and its mean value at the beginning of the study (*t* = 0) before the creation of the defect. For each difference that differs from 0, there is a 95% CI and the value of p for testing.

**Table 1:** This table shows mean and standard deviation of each variable (TRT group, CT group, difference, and SI), initially and after 1, 3, 9, and 18 months.

Month	TRT	CT	Difference	SI
0	273.73 ± 1.98	273.68 ± 2.00	0.05 ± 2.57	0.02 ± 0.94
1	294.18 ± 1.54	246.90 ± 1.12	47.28 ± 2.04	17.47 ± 0.74
3	292.43 ± 1.46	253.74 ± 1.72	38.69 ± 2.09	14.17 ± 0.78
9	276.11 ± 2.65	252.10 ± 2.90	24.01 ± 3.22	9.09 ± 1.23
18	275.50 ± 1.56	250.77 ± 2.02	24.73 ± 3.41	9.40 ± 1.31

**Table 2:** This table shows the range (minimum and maximum) of each variable each month.

Month	TRT	CT	Difference	SI
0	[270.7; 276.4]	[271.6; 277.9]	[−4.8; 3.4]	[−1.7; 1.2]
1	[291.6; 296.2]	[244.4; 248.1]	[43.5; 49.4]	[16.1; 18.2]
3	[290.7; 295.3]	[250.9; 256.7]	[34.0; 41.0]	[12.4; 14.9]
9	[272.6; 279.7]	[248.9; 256.3]	[20.5; 30.8]	[7.8; 11.6]
18	[273.2; 276.6]	[248.7; 253.2]	[20.0; 27.9]	[7.6; 10.6]

**Figure 4:**
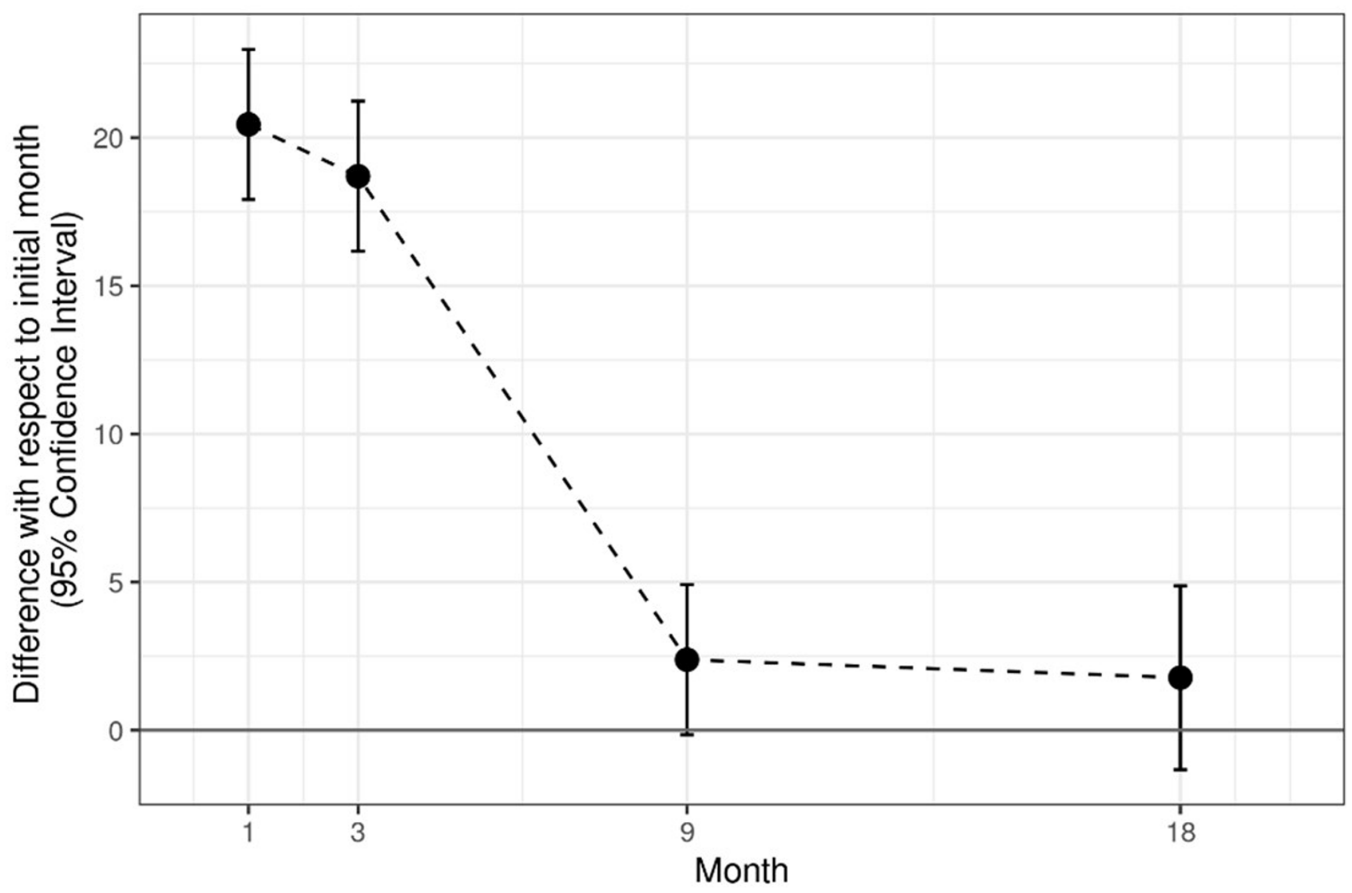
Analysis of PACI (TRT group).

**Figure 5:**
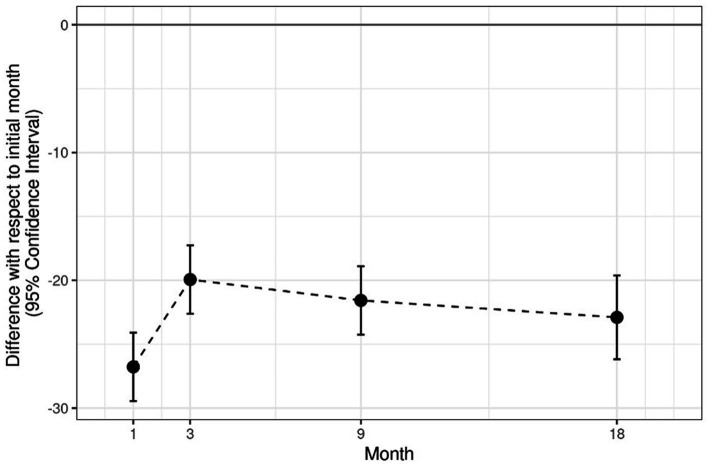
Analysis of RLS (CT group).

With regards to SI, significant differences (*p* < 0.0001) between the healthy limbs and the TRT limbs have been reported at 1-and 3-month follow up. However, no significant differences have been noticed between these two groups at 9-and 18-month follow up. Note that the SI in the TRT group increases significantly in months 1 and 3, but in months 9 and 18, its value remains stable (Table 3; Figure 4). Moreover, significant differences (*p* < 0.0001) between the CT group and HS have been shown at every study time, which means that the SI results obtained in the CT group showed statistically significant differences in all the analysis times (1, 3, 9, and 18 months; Table 4; Figure 5).

**Table 3:** Analysis of PACI (TRT group).

Comparison	Difference	CI95.	*p*
Month 1 – Month 0	20.45	[17.92, 22.98]	<0.0001
Month 3 – Month 0	18.7	[16.17, 21.23]	<0.0001
Month 9 – Month 0	2.38125	[−0.15, 4.91]	0.0759
Month 18 – Month 0	1.775	[−1.33, 4.88]	0.4995

**Table 4:** Analysis of RLS (CT group).

Comparison	Difference	CI95.	*p*
Month 1 – Month 0	−26.775	[−29.45, −24.10]	<0.0001
Month 3 – Month 0	−19.938	[−22.61, −17.26]	<0.0001
Month 9 – Month 0	−21.575	[−24.25, −18.90]	<0.0001
Month 18 – Month 0	−22.9	[−26.18, −19.62]	<0.0001

**Figure 6:**
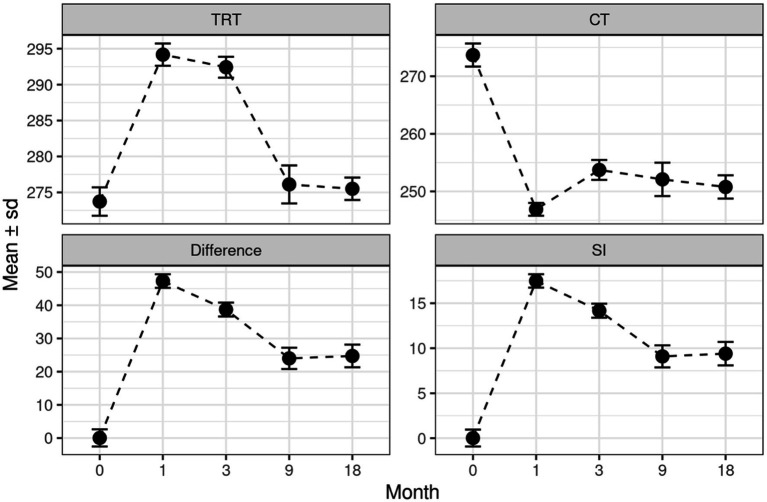
Shows mean and standard deviation of each variable (TRT group, CT group, difference, and SI), initially and after 1, 3, 9, and 18 months.

The model complies with the hypotheses of normality (Shapiro test *p* = 0.2362) and homoscedasticity (Levene test *p* = 0.1596; Tables 1, 2, 5; Figure 6).

**Table 5:** This table shows the global range of each variable.

TRT	CT	Difference	SI
[270.7; 296.2]	[244.4; 277.9]	[−4.8; 49.4]	[−1.7; 18.2]

### Macroscopic evaluation

The result of the analysis of these two scales shows better macroscopical articular cartilage regeneration in the TRT group compared to the CT group at 9 months. There were statistically significant differences when comparing the difference in the values of each macroscopic scale between TRT and CT at 9 months (Table 6).

**Table 6:** Results obtained for the different parameters in both ICRS and Goebel scales for stifles treated with PACI and RLS (±SD).

Scale	Macroscopic parameters	TRT		CT	
		9 months	18 months	9 months	18 months
ICRS	Repair	2.94 ± 1.00	3.75 ± 0.45	2.31 ± 0.95	3.19 ± 0.75
	Integration	2.38 ± 0.72	3.31 ± 0.60	2.12 ± 0.72	2.44 ± 0.73
	Appearance	2.12 ± 0.72	3.19 ± 0.66	1.75 ± 0.68	2.31 ± 0.79
	General	7.56 ± 2.28	10.12 ± 1.41	6.19 ± 2.10	7.94 ± 1.84
GOEBEL	Color	1.31 ± 0.60	0.31 ± 0.48	1.44 ± 0.81	1.19 ± 0.40
	Vessel	0.06 ± 0.25	0.00 ± 0.00	0.56 ± 0.73	0.31 ± 0.48
	Surface	1.44 ± 1.03	0.69 ± 0.79	2.00 ± 0.73	1.50 ± 0.89
	Filling	0.94 ± 0.85	0.50 ± 0.52	1.31 ± 0.70	1.00 ± 0.63
	Degeneration of adjacent articular cartilage	1.25 ± 0.45	0.44 ± 0.51	1.75 ± 0.86	1.50 ± 0.73
	General	5.00 ± 2.83	2.00 ± 1.71	7.00 ± 3.33	5.50 ± 1.86

**Figure 7:**
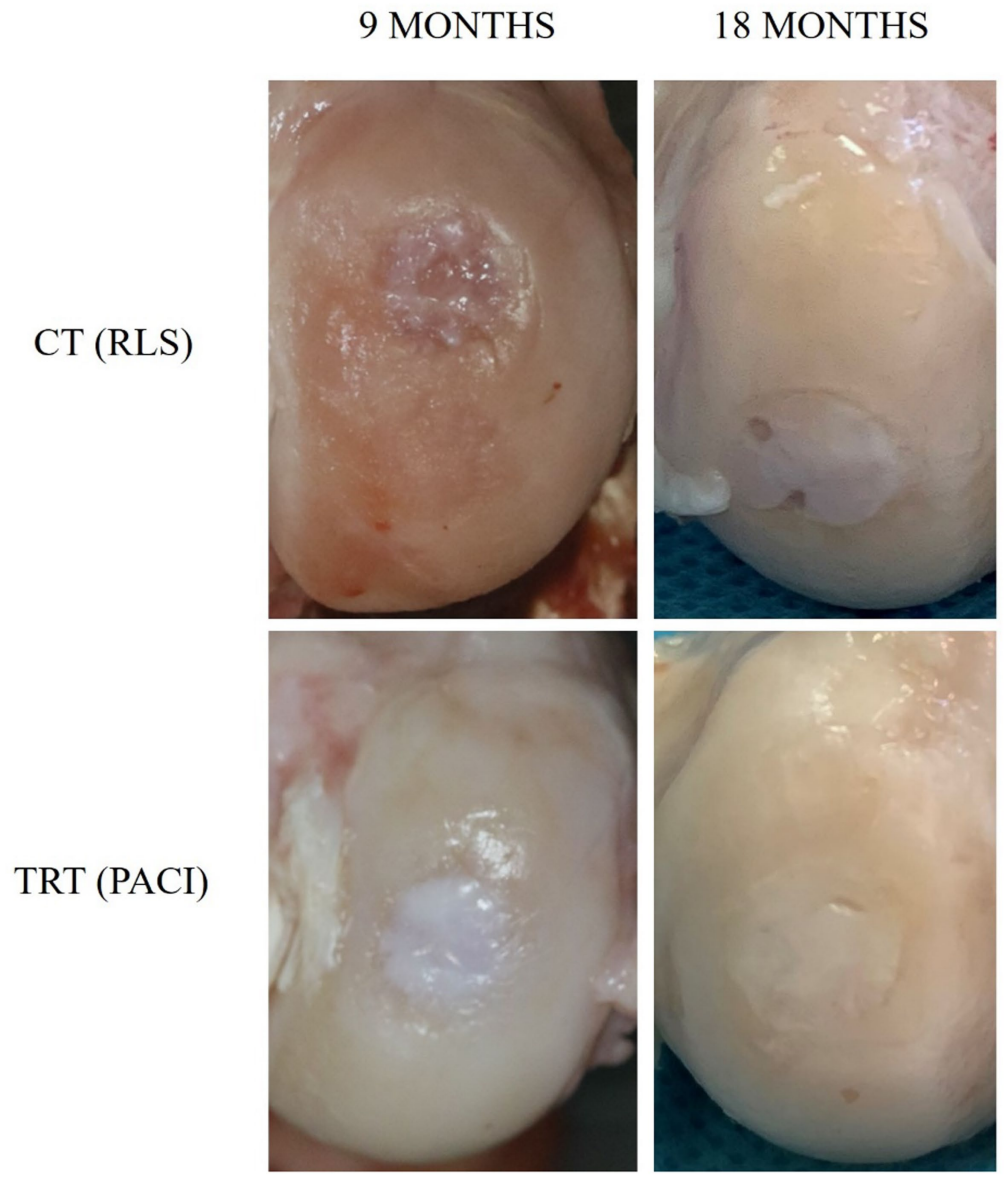
Macroscopic digital photographs of chondral defects: Group CT (RLS) 9 months and 18 months; Group TRT (PACI) 9 months and 18 months.

The mean scores for both macroscopical scales on each knee at 9-and 18-month follow up are compared in Table 7. There are no significant differences in any group between the scores obtained at 9-and 18-month follow up (Figure 7).

**Table 7:** The following table shows the mean difference (±SD) between the legs (treatments) at 9 and 18 months in general parameters of scales.

Month	Scale	CT	TRT	Diff CT – TRT (95%CI)	*P*	Shapiro.P
9	ICRS	6.19 (1.13)	7.56 (1.25)	−1.38 (−2.64, −0.11)	0.0401	0.2089
	GOEBEL	7.00 (1.85)	5.00 (1.56)	2.00 (1.17, 3.46)	0.0393	0.1314
18	ICRS	7.94 (0.84)	10.12 (0.57)	−2.19 (−4.97, 0.60)	0.0872	0.9028
	GOEBEL	5.50 (0.96)	2.00 (0.79)	3.50 (−0.19, 7.19)	0.0590	0.4295
Treatment	Scale	Month 9	Month 18	Diff 9–18 months (95%CI)	*P*	Shapiro.P
TRT	ICRS	7.56 (1.25)	10.12 (0.57)	−2.56 (−7.55,2.42)	0.1643	0.0615
	GOEBEL	5.00 (1.56)	2.00 (0.79)	3.00 (−0.42,6.42)	0.0508	0.1208
CT	ICRS	6.19 (1.13)	7.94 (0.84)	−1.75 (−5.64,2.14)	0.2845	0.7250
	GOEBEL	7.00 (1.85)	5.50 (0.96)	1.50 (−3.62,6.62)	0.4757	0.0561
Difference	ICRS	−1.38 (1.07)	−2.19 (1.07)	0.81 (−2.81,4.44)	0.6066	0.6951
	GOEBEL	2.00 (1.02)	3.50 (1.02)	−1.50 (−4.20,1.20)	0.2497	0.2708

## Discussion

In recent years, the application of previously minced small cartilage chips have achieved good results (32, 33). Some human and animal studies prove that there is better quality of repaired cartilage when minced cartilage treatments are used (34–36). This technique enhances the relocation of chondrocytes into the biomaterial and the subsequent cartilaginous extracellular matrix (ECM) deposition by these cells (37). Moreover, the IA application of PRP enhances cell migration and promotes chondrocyte viability, proliferation, and differentiation, which is essential during the chondrogenesis process (38, 39).

The present study aims to evaluate whether an aggregate of hyaline cartilage in a suspension of PRGF gel with the infiltration of PRGF IA in the stifle of a sheep with a chondral injury has satisfactory results. Currently, this treatment, called PACI, has been used in chondral injuries in humans, obtaining an optimal evolution of the patient evidenced by the improvement of clinical signs and imaging tests (15). In this sense, our working hypothesis is that the application of an autologous-made matrix involving hyaline cartilage “chips” mixed with a PRGF-based clot, together with the IA infiltration of PRGF in sheep with a chondral stifle injury, will achieve an optimal clinical evolution. The effectiveness was assessed by functionality tests and objective force platform gait analysis. In addition, we believe that this new biological therapy produces the repair with hyaline cartilage with similar characteristics to the healthy one, which will be evidenced with macroscopic study.

In our study, the healthy hyaline cartilage used in the TRT group was cut into 1–2 mm^3^ fragments. It is important to consider the degree of fragmentation of the cartilage chips because it influences the amount of ECM production (40). Similar to our study, Alcaide-Ruggiero et al. fragmented the cartilage particles into cubes of 1–2 mm^3^, obtaining promising results in cartilage repair (17). Moreover, Anitua et al. have recently published that the mean size of the cartilage fragments should be 1.7 mm^2^ (41).

In reference to the macroscopic study at 9 months, the TRT group showed significantly better results than the CT group (Table 6). In accordance with our results, Alcaide-Ruggiero et al. have recently published a study in which 16 sheep were included. The animals were divided into two groups (*n* = 8). In the first group, left stifles were treated with RLS, while right stifles were treated with PACI+PRP. In the second group, left knees were treated with hyaluronic acid (HA), and PACI+PRP was used for the right knees. The sheep were euthanized at 9 and 18 months after surgery, and the macroscopic evaluation showed that PACI+PRP patients had better macroscopic appearance than the RLS and HA animals, especially at 18-month follow up. This group has recently published the use of PACI+PRP treatment in knee chondral defects, showing an improvement in the repair of the cartilage process, with the presence of mature hyaline cartilage that shows better functional characteristics (16, 17). In the same line, Dominguez et al. conducted a study with sheep to analyze the repair tissue macroscopically, histologically, and immunohistochemically after the application of the PACI treatment. The results of this study showed that this technique was able to repair hyaline articular cartilage with an adequate presence of type II collagen and little presence of type I collagen. The authors observed that this novel treatment promoted chondrogenesis and regenerated hyaline cartilage at 6-month follow up with closely normal macroscopic ICRS assessment (2). Similar results were obtained by Milano et al. in which the ICRS macroscopic scale was used to assess full-thickness chondral lesions of the medial femoral condyle in 15 sheep. All sheep were treated with a microfracture surgical technique, but better ICRS scores were obtained in animals additionally treated with a fibrin glue gel implant combined with PRP than in the ones only treated with microfractures or microfractures together with PRP injections (13).

Limb function was also assessed in our study. Our results showed that at 1-month follow up, there were significant differences between the TRT group and the HS group, because the animals supported more weight on the treated limb than on sound limbs. This could be explained because both limbs were subjected to a surgery; therefore, more weight was loaded onto less painful limbs to compensate for the hurt limb. In the same way, at 3-month follow up, significant differences between the TRT group and the HS were reported. However, at 9-and 18-month follow up, the TRT group and the HS group were reported, meaning the TRT limb supported the same weight as the HS limb. On the contrary, the SI results obtained in the CT group showed significant differences with the HS group during all the study times (1, 3, 9, and 18 months), meaning the sheep never achieved the same limb function after surgery. These results cannot be compared with previously published results because, to our knowledge, no force platform analysis has been conducted in sheep. However, some research in dogs have extensively studied the compensatory mechanisms between sound and affected limbs (13, 42–45).

In humans, the first publication of two patients with full-thickness chondral lesions treated with PACI were professional soccer players (14). The study concluded that pain relief was satisfactory, and the players returned to the same level of functionality they had prior to the injury. These results are similar to the ones obtained in our study, where stifles treated with PACI also returned to full functionality, objectively generating force loads equal to those of a healthy limb. Also, in the same study, the authors reported that in a second-look arthroscopy, they observed a similar appearance and consistency on palpation of the new cartilage to the immediate healthy articular cartilage. However, it was not possible to analyze the repair tissue histologically and immunohistochemically (14). The same research group confirmed good clinical and functional results in a clinical study performed on 15 patients with full-thickness knee cartilage defects or osteochondritis dissecans based on MRI findings (quality and quantity of cartilage repair) in 2020. Actually, this research group has used PACI in more than 150 patients with chondral defects in different joints for over 5 years. They have reported good clinical, functional, and MRI-based results (15).

The good results reported in our study and in research previously published regarding histology and functional outcomes after PACI treatment could be explained by the results obtained by Anitua et al. in an *in vitro* research study recently published. The authors concluded that cartilage fragments embedded into the three-dimensional PRGF scaffold contained viable chondrocytes that were able to migrate into the fibrin network and proliferate and synthesize the extracellular matrix after the second week of *in vitro* culture (41).

One of the purposes of our study is to demonstrate that PACI is a therapy that can replace other repair techniques for articular cartilage injuries, for instance ACI. The ACI technique requires *in vitro* expansion of the patient’s chondrocytes, which requires about 4 weeks, so cell dedifferentiation may occur. In addition, this technique can present drawbacks, such as a high cost for the production of cells, different surgical interventions, and a long time to return to functional activity (29). These types of complications raise the importance of creating new regenerative and reparative therapies for chondral defects, which manage to obtain normal hyaline cartilage. In recent years, other therapies have been investigated, such as infiltrations with PRP and MSCs. MSCs synthesize trophic factors with regenerative functions, and they are able to differentiate into cartilaginous and osseous cells, favoring articular cartilage regeneration (46) The IA infiltration of this regenerative therapy facilitates the expression of molecules with chondrogenic and anti-inflammatory activity, and it has been demonstrated that they generate an environment that favors the recruitment and activation of stem cells on site, enhancing the repair of articular cartilage (47). Regarding the treatment of articular cartilage injuries with PRP (which we combined in the present study with cartilage chips), different studies demonstrated that PRP regulates cartilage repair by stimulating the secretion of type II collagen, aggrecans, or proteoglycans; stimulating the chondrogenic differentiation of MSCs; and proliferating synoviocytes. Additionally, PRP reduces the catabolic effects of cytokines, such as IL-1, and of proteolytic enzymes, like matrix metalloproteinases (27, 46, 48). We must consider that it is especially valuable to provide alternative, minimally invasive therapeutic modalities that might significantly halt the disease progression in patients with chondral lesions. The main finding of this study is that this new treatment modality is able to regenerate hyaline articular cartilage. The present research provided superior functional outcomes with a better macroscopical appearance of the cartilage repair tissue in sheep. Bioregenerative therapies have proven to be a better option over other treatments due to their therapeutic potential. In recent years, these therapies have been proposed as a promising alternative or a complementary therapy to conventional treatments, with the objective of improving tissue regeneration. The main limitation of this study is that our sheep underwent the surgical procedure in both right and left stifle. Ideally, only one stifle per sheep should be operated on in order to compare the healthy stifle with the damaged stifle within the same animal. Moreover, sheep did not follow any kind of movement restriction, thus the sheep could have suffered a matrix detachment during the first days after PACI implantation.

## Conclusion

Our results showed that PACI-treated limbs obtained values equivalent to those obtained by healthy limbs at 9-and 18-months follow up. Furthermore, the macroscopical analysis of the postmortem lesions showed better scores in the TRT group than in the CT group at 9-month follow up.

It can be concluded that PACI treatment is a good therapeutic option for full-thickness chondral lesions, as excellent functional recovery has been observed in a sheep model. In addition, the technique is safe because it uses autologous tissue with no probability of refusal by the patient. Moreover, the technique is easy to reproduce and does not require a high economic investment. Nonetheless, further research is required to compare PACI with other treatments and to assess the efficacy of this therapy at longer follow-up periods.

## Data availability statement

The raw data supporting the conclusions of this article will be made available by the authors, without undue reservation.

## Ethics statement

The animal study was approved by CEU CARDENAL HERRERA UNIVERSITY. The study was conducted in accordance with the local legislation and institutional requirements.

## Author contributions

JC-P, JV-G, PP-G, and RC-B contributed to conception and design of the study. PP-G, MR-Z, ED-G, JV-G, JC-P, and JS-J analyzed data and wrote the manuscript. AS performed statistical analysis. PP-G, MR-Z, ED-G, JV-G, JC-P, and JS-J wrote the first draft of the manuscript. PP-G, JC-P, ED-G, BC-S, MR-Z, ÁH-G, JS-G, AR-M, LM-P, and MT-T performed the experiment. All authors contributed to the article and approved the submitted version.
